# Structured Light Plethysmography for Non-Invasive Assessment of Respiratory Pattern in Spinal Muscular Atrophy Type 1

**DOI:** 10.3390/jcm12247553

**Published:** 2023-12-07

**Authors:** Noemi Brolatti, Federica Trucco, Marta Ferretti, Chiara Avanti, Paola Tacchetti, Chiara Panicucci, Pasquale Striano, Carlo Minetti, Claudio Bruno, Marina Pedemonte

**Affiliations:** 1 Paediatric Neurology and Muscle Disease Unit, IRCCS Istituto Giannina Gaslini, 16147 Genova, Italy; noemibrolatti@gaslini.org (N.B.); martaferretti@gaslini.org (M.F.); chiara.avanti@gmail.com (C.A.); paola.tacchetti@gmail.com (P.T.); 2 Department of Neuroscience, Rehabilitation, Ophthalmology, Genetics, Maternal and Child Care, University of Genova, 16132 Genova, Italypstriano@unige.it (P.S.); minettic@unige.it (C.M.); claudiobruno@gaslini.org (C.B.); 3 Centre of Translational and Experimental Myology, IRCCS Istituto Giannina Gaslini, 16147 Genova, Italy; chiarapanicucci@gaslini.org

**Keywords:** spinal muscular atrophy type 1, structured light plethysmography (SLP), CHOP-INTEND

## Abstract

Background: Spinal muscular atrophy (SMA) type 1 is a severe condition leading to early respiratory failure. Treatment options have become available, yet respiratory outcome measures in SMA type 1 are limited. The aim of this study was to assess the respiratory pattern in SMA type 1 patients via structured light plethysmography (SLP). SLP measures the thoraco-abdominal movements by projecting a light grid onto the anterior thoraco-abdominal surface. Methods: Cross-sectional study of consecutive children with SMA type 1. All children underwent motor assessment (CHOP-INTEND) and one-minute tidal breathing recording by SLP in supine position while self-ventilating in room air. The *Respiratory rate*, the abdominal vs. chest contribution to breath (*Relative Expired Abdomen%*, *Relative Expired Chest%*) and the severity of thoraco-abdominal paradox (*Phase Angle*) were acquired. Results: Nineteen patients were included, median (IQR) age 2.3 years (1.4–7.9). Their respiratory pattern captured via SLP showed a raised median (IQR) respiratory rate per age of 33.5 bpm (26.6–41.7), a prevalent abdominal contribution to tidal breathing with median (IQR) *Relative Expired Abdomen* 77% (68–90) vs. *Chest* 23% (10–32). Thoracoabdominal paradox was detected (median *Phase Angle* 48.70°) and its severity correlated negatively with CHOP-INTEND (r −0.8, *p* < 0.01). Conclusions: SLP captured and quantified the respiratory features of infants and children with SMA type 1.

## 1. Introduction

Spinal muscular atrophy (SMA) is an autosomal recessive neurodegenerative disorder with reported incidence from 1:6000 to 1:15,000 live births identified at newborn screening [1,2]. It is characterized by progressive muscle wasting due to motor neuron degeneration caused by mutations of the *SMN1* gene leading to the lack of full-length SMN protein needed for motor neuron survival. A paralogue gene, *SMN2*, which differs from *SMN1* by a mutation in the splice acceptor site of intron 7, is responsible for the production of only around 10% of functional SMN protein. The number of copies of *SMN2* correlates, albeit not invariably, with the severity of the disease [3].

SMA encompasses a wide range of phenotypes classified by age of onset and maximum motor function achieved. At the most severe end of the clinical spectrum, SMA type 0 and 1 present either at birth or within the first months of life, whilst the milder types have onset later in childhood (type 2 and type 3) or adulthood (type 4) [4,5].

SMA type 1 is clinically characterized by severe hypotonia and early onset respiratory failure which requires either non-invasive (NIV) or invasive ventilatory support [6,7]. Affected children are more prone to respiratory infections and cough impairment due to severe respiratory muscle weakness with patients historically dying before two years of age [8,9].

The hallmarks of respiratory involvement in SMA type 1 are the weakness of intercostal muscles in the chest with a relative sparing of the diaphragm, leading to the so-called paradoxical breathing and the narrowing of the ribcage [6,10,11].

In the last few years, the combination of a pro-active multidisciplinary approach [4,12] and the availability of disease-modifying treatments (DMT) such as antisense oligonucleotide (nusinersen) [13], small molecule (risdiplam) [14] and gene therapy (Onasemnogene abeparvovec) [15,16] approved for SMA type 1 has led to an overall improvement in patients’ respiratory status with subsequent longer survival [17,18,19].

In the context of these evolving phenotypes, objective and repeatable tools to assess the respiratory function of patients with SMA type 1 are needed, particularly considering the young age and the lack of cooperation of these children. Promising attempts have been carried out via Optoelectronic Plethysmography (OEP), a tool to measure motion and volumes of ribcage and abdomen via the displacement of markers applied to the patient’s skin. OEP has provided remarkable insights into the respiratory features of children with SMA type 1 and the role of treatments, such as nusinersen. However, the setup of OEP requires an ad hoc research facility and experienced professionals [11,20,21].

Structured light plethysmography (SLP) is a non-invasive, light-based method of assessing patients’ breathing pattern by projecting a grid of light onto the anterior thoraco-abdominal wall. The axial displacement of the light grid, recorded by digital video cameras, measure the thoraco-abdominal movement during tidal breathing over a defined amount of time (up to 5 min). The post-processing analysis of the recording provides additional outputs such as respiratory times and other indexes of breathing pattern such as the contribution of chest and abdomen to each breath and the degree of thoraco-abdominal synchrony [22,23]. SLP was feasible in newborn [24], non-cooperative young children [25,26] and in young boys with Duchenne Muscular Dystrophy [27].

The main aim of this study was to assess the feasibility of SLP in capturing the respiratory pattern of a cohort of infants and young children with SMA type 1 naïve from any disease-modifying treatment. The secondary aim was to correlate the respiratory output obtained by SLP with patients’ clinical features and motor function.

## 2. Materials and Methods

### 2.1. Study Population

This was a cross-sectional study enrolling consecutive children (age < 18 years) with SMA type 1 referred to the Paediatric Neurology and Muscle Disease Unit, Istituto Giannina Gaslini, Genoa, from June 2016 to May 2017. The study protocol was approved by the Regional Ethic Committee—San Martino Hospital, Genoa (REC 377REG2015), and was carried out according to the Helsinki Declaration. Written consent was obtained from patients’ parents/caregivers of all children before the enrolment in this study.

Children with a confirmed genetic diagnosis of SMA and clinical features consistent with SMA type 1, namely onset of symptoms before 6 months of age and inability to sit unsupported, and on ventilatory support for less than 24 h/day, either invasive or non-invasive, were included. Patients acutely unwell at the time of recording and children with severe scoliosis causing chest/abdominal asymmetry were excluded.

### 2.2. Study Protocol

All patients underwent a standardised protocol of assessments to ensure consistency. Anthropometrics (height, weight, chest circumference) were collected as a first step by the same paediatric nurse. A five-minute SLP recording was performed by the same operator. Finally, the same trained and certified neuromuscular physiotherapist performed motor assessments consisting of Hammersmith Infant Neurological Examination (HINE) and Children’s Hospital of Philadelphia Infant Test of Neuromuscular Disorders (CHOP-INTEND).

#### 2.2.1. Anthropometrics and Clinical Features

Patients’ length was recorded in supine position with a stadiometer and weight as per routine clinical assessment. Length (cm), weight (kg) and BMI (kg/m^2^) were expressed as absolute values and percentiles based on the World Health Organization (WHO) infant charts for children between 0 to 2 years of age and children older than 2 years of age. Chest circumference was measured at the interaxillary line. The presence of scoliosis was recorded.

The regular use of cough assist device at home, the type of respiratory support (Invasive vs. non-invasive) and hours of respiratory support per day were collected for all patients. Feeding support information, namely the use of gastrostomy, was collected.

The respiratory rate for each patient was assessed both visually and by SLP and was compared to normal respiratory rate published on age-matched healthy children [28].

#### 2.2.2. Protocol of Assessments of Respiratory Pattern by Structured Light Plethysmography

The assessment of the breathing pattern was carried out with SLP PneumaCare Thora3DI^®^ device (Cambridgeshire, UK) combined with the light projector Thora 3DI^®^. The child’s breathing pattern was measured using a grid of light, tracked by a digital vision system of two high-speed cameras.

Each patient underwent five-minute recording of tidal breathing in the supine position in room air. All recordings were carried out with children bare chested. Ventilatory support was removed at least 30 min before the recording in those patients using ventilation daytime and overnight.

**Figure 1:**
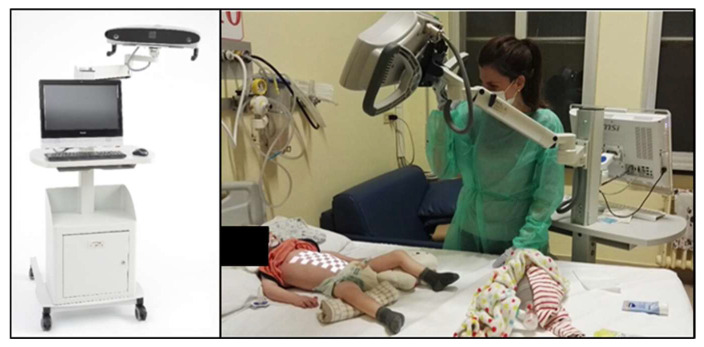
PneumaCare Thora3DI^®^ System device and acquisition of respiratory pattern in a child with SMA type 1. Protocol of acquisition of breathing pattern via structured light plethysmography.

Three SLP grid sizes were available (small, medium and large) according to the number of squares (14 × 10, 12 × 8, 10 × 6). Each square of the grid contributed equally to the signal. For all recordings, the medium SLP grid size was used. The SLP grid was centred according to the patients’ sternum xiphoid process and aligned with this placement. The SLP projector was adjusted before the recording to consistently capture the same regions in all patients. The “chest” was defined as spanning from the clavicles to the sternum xiphoid process and the “abdomen” the region from the sternum xiphoid process to the anterior iliac crests. Patients laid at a distance of up to 1 m from the SLP projector. The distance to the patient was adjusted to maintain neat margins of the light grid. Sampling rate was 30 Hz (Figure 1).

Immediately after the recording, the quality of traces was checked. If the recording was deemed suitable for analysis by the same operator (M.F), it was saved and stored in the system for processing. A post-recording phase consisted of selecting one out of the five minutes recorded based on the quality of the traces, always avoiding the first and last minute of recording.

**Figure 2:**
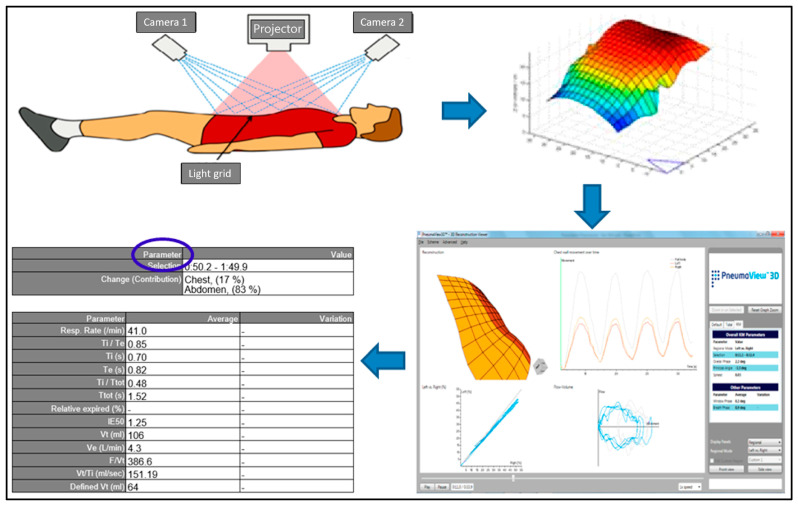
Data acquisition, recording and processing via PneumaView™ 3D. The PneumaCare Thora3DI^®^ projected a light grid on the chest of the patient lying in supine position (**top left**). The displacement of the thoraco-abdominal light grid was three-dimensionally reconstructed by the PneumaView™ 3D (**top right**). After the recording, the PneumaView™ 3D provided a visual (**bottom right**) and numeric (**bottom left**) output of the information available for the analyses. Blue arrows indicate the sequence of breathing pattern recording and data acquisition. Blue circle indicates the relevant parameters provided by the PneumaView™3D.

The data gathered was analysed via the built-in software (PneumaView™ 3D, V1.0). PneumaView™ 3D provides a tri-dimensional static and in-motion reconstruction of the patient’s chest and abdomen within the recorded time and a graphical representation of breathing pattern with chest and abdomen breathing movements represented as sinus waves (Figure 2).

The synchrony of chest and abdomen during each breath within the recorded time was expressed as *Phase Angle* (*Overall phase*) defined as the phase difference between chest and abdominal trace.

When chest and abdomen move in perfect synchrony the *Phase Angle* is zero (i.e., no difference exists between the phase of movement of chest and abdomen, and both are in inspiration or expiration at the same time). As a degree of asynchrony appears (out-of-phase breathing), the absolute value of the *Phase Angle* increases up to the event of complete asynchrony (paradoxical breathing) expressed as a *Phase Angle* of 180° (i.e., chest and abdominal movements are completely out of phase and one region is in expiration when the other is in inspiration and vice versa).

In this study, the thoraco-abdominal asynchrony was defined as a value of *Phase Angle* between 0° and 180° and paradoxical breathing as a *Phase Angle* equal to 180°. The degree of relative contribution to the breathing movements within the recorded time of the chest (expressing the degree of motion of intercostal muscles) and the abdomen (expressing the motion of diaphragm) were expressed as *Relative Expired Chest* % and *Relative Expired Abdomen* %.

Considering the thoraco-abdominal region as being composed of two compartments, the relative contribution of any compartment was quantified as percentage of the total displacement. In this study, the *Relative Expired Chest* % and *Relative Expired Abdomen* % were defined as the ratio between the range of movement of chest and abdomen considered separately during expiration and the total thoraco-abdominal range of movement during expiration averaged over all breaths recorded.

The *Respiratory rate* (*RR*) expressed as breath per minute, was also acquired during the SLP protocol of acquisition as the number of breaths per minute averaged over all breaths recorded. The respiratory rate is an indicator of the breathing effort the patients need to make to maintain adequate levels of oxygen saturation and carbon dioxide.

The *Rapid Shallow Breathing* expresses the ability to move adequate volumes of air with each breathing cycle. The *Rapid Shallow Breathing Index* (RSBi) is mostly used in intensive care settings as predictor for extubation [29]. In this study, the RSBi was calculated as the ratio between patients’ respiratory rate and tidal volume (Vt) both derived from the same SLP recording.

A standard tidal volume (Vt) of 8 mL/kg was entered manually based on patient’s weight before each recording according to the manufacturer’s instructions given volumetric measurements were not a direct output of SLP. The software provided an estimated average Vt after the recording [22].

The median (IQR) value of *Phase Angle* (°), *Relative Expired Abdomen %*, *Relative Expired Chest %*, *Respiratory rate* and *RSBi* over a single SLP recording session were collected for all patients and were correlated with patients’ anthropometrics and motor functional tests.

#### 2.2.3. Motor Assessments

Motor assessments consisted of the Hammersmith Infant Neurological Examination (HINE), which assesses children’s motor developmental milestones [30], and the Children’s Hospital of Philadelphia Infant Test of Neuromuscular Disorders CHOP-INTEND, a global motor function assessment created ad hoc for children with SMA. Both assessments were previously validated in children with SMA type 1 [31].

### 2.3. Statistical Analyses

Statistical analyses were conducted using IBM SPSS^®^ version 24. Descriptive statistics was used to describe study population features and anthropometrics. Distribution has been assessed for all the variables. However, given the relatively small sample size, non-parametric analyses were used. Descriptive data were expressed as median and interquartile range (25th–75th centile, IQR). Wilcoxon test has been used to compare the respiratory rate in our population with the respiratory rate of age-matched normal data. Finally, the correlations between respiratory and either clinical variables/anthropometrics or motor function assessments were performed by Spearman rank correlation.

## 3. Results

### 3.1. Study Population

Overall, 19 children with SMA type 1, 13 male (68%), were included. Their median age was 2.3 years (IQR 1.3–7.9). Further details regarding the study population are summarised in Table 1 and Table 2.

### 3.2. Respiratory Pattern Captured by SLP

A five-minute SLP recording was feasible in all patients in the supine position, comfortably placed in a hospital bed in an outpatient room. As per the study protocol, after the quality check of the quality of the acquisition, only a one-minute recording was kept for analysis.

A summary of the output recorded from one-minute post-recording is presented in Table 3.

**Figure 3:**
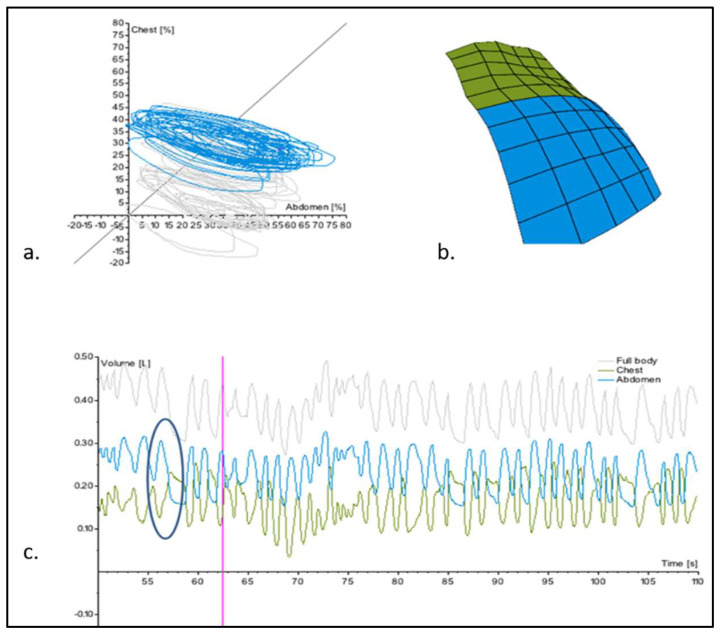
Visual output of a patient’s breathing pattern provided by PneumaView™ 3D. (**a**) Konno–Mead loop showing the prevalent contribution (percentage) of abdomen versus chest to each recorded breath. When the contribution is equally distributed, the main axis of the loop is parallel to 45° and the Principal angle is 0°. In the loop represented here, the Principal angle (the angle between the 45° line and the principal axis of the Konno–Mead curve) is negative showing a prevalent abdominal breathing component. (**b**) Three-dimensional reconstruction of the patient’s chest (green) and abdomen (blue) during quiet breathing. (**c**) Sine wave representation of chest (green) and abdominal (blue) motion during quiet breathing. The breathing pattern is characterised by a thoraco-abdominal phase shift and paradoxical breathing (blue circle). Pink line indicates the recording (60 s) saved for analysis of breathing pattern.

Figure 3 shows an example of the visual outputs provided by SLP for one of the patients enrolled in this study.

The analysis of the acquisition showed that all patients had a positive *Phase Angle*, suggesting the presence of a certain degree of thoraco-abdominal asynchrony. The absolute value of the *Phase Angle* ranged from 34.3° to 144.4°, meaning that none of the patients in the study population had either a complete in-phase or complete paradoxical breathing.

The median value of the *Phase Angle* was 48.7° (IQR 41.6–63.2), which is higher than the reference values (11.8°) for normative data for age-matched healthy children [28].

In the study population, the contribution of the abdomen to each breath was higher than the contribution of the ribcage. The median value of the *Relative Expired Abdomen* was *77%*, while the median value of the *Relative Expired Chest* was *23%* (*p* < 0.05).

The median value of respiratory rate was 33.5 (26.6–41.7) bpm, which was higher than normative data for age-matched healthy children [28].

Finally, the median value of the Rapid Shallow Breathing index (RR/Vt (l)) was 342.1 (236.5–457.9), which is considered elevated, given that any value above 105 is considered associated with a poorer prognosis of respiratory failure [29].

### 3.3. Correlation between the Respiratory Pattern Derived by SLP and Patients’ Clinical Features

The output derived from SLP recording was correlated to patients‘ chest circumference, BMI and hours of ventilatory support per day.

Patients with higher *Respiratory Rate*, longer ventilator requirement per day and smaller chest circumferences had a trend towards a higher contribution of the abdomen to the breathing (expressed as *Relative Expired Abdomen %*), but a significant correlation was not found.

Patients with a smaller chest circumference had a higher value of the Rapid Shallow Breathing index, *RSBi* (r −0.7, *p* < 0.01). The degree of *RSBi* did not correlate with the patients‘ ventilator requirement.

### 3.4. Correlation between Respiratory Pattern and Motor Function

The degree of thoraco-abdominal asynchrony *(Phase Angle)* strongly negatively correlated with CHOP-INTEND (r −0.8, *p* < 0.01). Patients’ daily hours of ventilation strongly negatively correlated with HINE (r −0.8, *p* < 0.01), whilst a trend was observed with the CHOP-INTEND.

**Figure 4:**
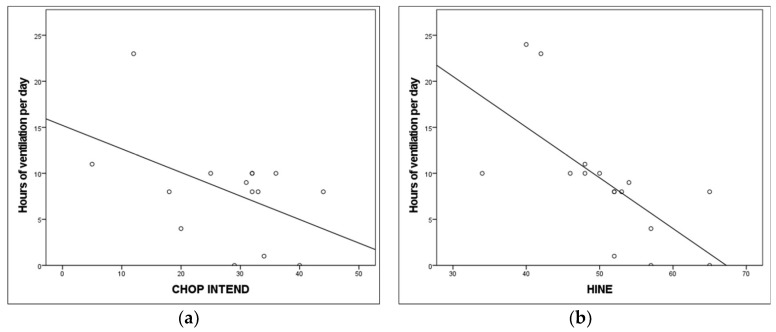
Correlation between ventilator requirement and motor function in SMA type 1. (**a**) Children’s Hospital of Philadelphia Infant Test of Neuromuscular Disorders (CHOP-INTEND). (**b**) Hammersmith Infant Neurological Examination (HINE).

Figure 4 summarizes the correlation between the severity of patients’ motor function and their requirement for ventilatory support expressed as hours per day.

Patients’ respiratory rate, the degree of Rapid Shallow Breathing and the relative contribution of the abdomen to the breathing did not correlate with either CHOP-INTEND or HINE.

## 4. Discussion

This is the first attempt to use structured light plethysmography (SLP) in the assessment of respiratory pattern in a population of infants and young children with SMA type 1. We showed that SLP did not require patients’ collaboration, was feasible and well tolerated in all children as young as 12 months of age and allowed the simultaneous recording of multiple respiratory parameters.

Taken together, the respiratory pattern recorded by SLP was characterized by well-recognised hallmarks of SMA type 1 which are (i) rapid and shallow breathing, (ii) predominantly abdominal breathing associated with (iii) a thoracoabdominal asynchrony.

Each one of these respiratory features was correlated in our study with the motor and anthropometric parameters of the 19 patients providing interesting and novel insights.

Those children with the highest rate of shallow breaths were those with the narrowest (bell-shaped) chest circumference. This confirms that, in SMA type 1, breathing small tidal volumes of air is compensated by a higher respiratory rate to ensure normal gas exchange. The absolute chest circumference, used in this study as an index of narrow chest, is affected by patients’ age and growth. A “bell-shaped chest index”, defined as the ratio between the distance of the two anterior axillary lines and the distance between the tenth costal cartilages, has been proposed as a surrogate measurement of narrow chest in SMA patients but was not available at the time of this data collection [32].

Secondly, the predominant abdominal contribution to breathing has been described in SMA [11] as the expression of the atrophy of intercostal muscles associated with the relative sparing of the diaphragm. In our cohort, we showed that children with a lower abdominal contribution to breathing, i.e., those whose intercostal muscles were still contributing to the breathing, required less ventilator support over 24 h.

Finally, those patients with a more severe thoraco-abdominal asynchrony during breathing due to the more severely affected inter-costal respiratory muscles and expressed as a positive *Phase Angle* at SLP, were also the most affected motor-wise (i.e., scoring less at CHOP-INTEND).

This study showed that a non-invasive tool such as SLP can be reliably added to the clinical respiratory assessment of young non-cooperative children. Objective measures of patients’ respiratory pattern could complement the assessments of motor function in SMA to help clinicians, for instance, in the early identification of children who would require ventilatory support. In research settings, SLP could support the acquisition of new outcome measures in the context of clinical trials.

Recent cross-sectional studies have evaluated new tools for the assessment of respiratory function in children with SMA type 1. Lo Mauro et al. [11] assessed the breathing pattern in these group of patients using Optoelectronic Plethysmography (OEP). In keeping with their findings, our data confirmed the relationship between shallow and paradoxical breathing with a reduced chest contribution to each breath as specific respiratory features of SMA type 1. Here, we additionally showed that the severity of patients’ paradoxical breathing did correlate with the severity of the ribcage narrowing, expressed as chest circumference and with patients’ ventilator requirement per day.

Finkel et al. [33] evaluated patients’ thoraco-abdominal asynchrony via respiratory inductance plethysmography. Consistently with our results, most of their patients with SMA type 1 (6 out of 7) had a nearly complete paradoxical thoraco-abdominal motion with a *Phase Angle* as high as 140°.

Other works proposed new tools to non-invasively assess respiratory function in SMA [34]. Lung Clearance Index (LCI) is a technique that assesses lung volumes by measuring the time to washout a set amount of inert gas from the lungs [35,36]. It was trialled in young patients with SMA type 1 but was not suitable as it requires patients’ collaboration. Kapur et al. [34] also trialled the Forced Oscillation Technique (FOT), a technique that measures the resistance of airway by the application of oscillations to patient airways during patients’ quiet breathing [37]. It was shown to be feasible in children with SMA (3 patients SMA type 1) as young as 3 years of age but was not found to correlate with the patient’s use of NIV.

Despite its novelty and its prospective design, this study has several limitations, the main one being the relatively small sample size and the lack of an age-matched control population. Also, the reliability and repeatability of SLP still need to be tested in wider populations of neuromuscular disorders. SLP could be potentially added to longer observational studies looking at SMA types 2 and 3 and aimed to detect any difference in the respiratory pattern between motor functional groups and their progression over time. Prospective studies in patients with SMA type 1 will assess the effects of DMT on patients’ respiratory function assessed by SLP.

In conclusion, the assessment of the respiratory pattern using structured light plethysmography was feasible in young, non-cooperative children with SMA type 1 naïve from disease-modifying treatments included in this study (median age 2.3 years). The analysis of SLP outputs showed the selective contribution of chest and abdominal muscle groups to the breathing pattern showing Rapid Shallow Breathing, a predominant abdominal contribution to breathing and a thoraco-abdominal asynchrony. The severity of thoraco-abdominal asynchrony was higher in weaker patients as assessed via CHOP-INTEND. Larger prospective studies on patients with SMA need to be carried out to evaluate the capability of SLP in capturing changes over time and after treatment.

## Figures and Tables

**Table 1:** Clinical features of study population.

*N* = 19	*N* (%)	Median Age (IQR)
Male, *n* (%)	13 (68%)	
Age (years)		2.3 (1.3–7.9)
Age of onset (years)		0.3 (0.2–0.4)
Age at diagnosis (years)		0.6 (0.3–0.9)
*SMN2* copies		
- 1	0 (0%)	
- 2	19 (100%)	
- other	0 (0%)	
Use of cough assist (yes)	19 (100%)	
Age starting ventilatory support (months)		10.5 (5.5–24)
Ventilatory support (yes), *n* (%)	17 (89%)	
- Invasive	4	
- Non-Invasive	13	
Ventilation requirement (yes), *n* (%)		
- 1–8 h/day	6	
- 9–16 h/day	6	
- 17–23 h/day	5	
Orthopaedic features		
- Scoliosis (yes)	10 (53%)	
- Joint contractures	14 (74%)	
Feeding		
- Oral	6	
- Nasogastric tube	3	
- Age at NG (months)		9 (5–14.5)
- Gastrostomy	10	
- Age at gastrostomy (months)		23 (11–26)
Comorbidities		
- Interatrial septum defect	2	
- Pre-term birth	2	
- Oculocutaneous albinism	1	
- Adenotonsillar hypertrophy	1	
Treated with disease modifying therapies	0	
Concomitant therapies		
- Salbutamol	4	
CHOP-INTEND	15	32 (20–34)
HINE	16	52 (46–57)

Abbreviations: IQR: Interquartile range; CHOP-INTEND: Children’s Hospital of Philadelphia Infant Test of Neuromuscular Disorders; HINE: Hammersmith Infant Neurological Examination.

**Table 2:** Anthropometric data of study population.

*N* = 19	*N* (%)	Median (IQR)	WHO Percentile
Height (cm)	16 (84%)	89.5 (75–118.3)	9th–25th percentile
Weight (kg)	16 (84%)	10.2 (7.2–17.9)	<3rd percentile
BMI (kg/m^2^)	16 (84%)	13.2 (11.5–14.7)	<3rd percentile
Chest circumference (cm)	11 (58%)	47.5 (41–57.5)	

Abbreviations. IQR: Interquartile range; WHO: World Health Organization; BMI: Body Mass Index.

**Table 3:** Summary of respiratory parameters acquired by one-minute SLP recording, reference values per age range and clinical meaning.

SLP Parameters	Median (IQR)	Reference Value (Healthy Subjects)	Clinical Expression of Respiratory Pattern in SMA Type 1
Respiratory rate (bpm)	33.5 (26.6–41.7)	27 CI 95% (26–28) age 2.3 [28]	Increased RR
Vt (mL) pre-imposed (8 mL/kg)	79.0 (57–140.5)		
Vt (mL) *	95.5 (56.2–120.3)		
Vt (mL)/Vt (mL) pre-imposed (%)	94 (75.6–117.6)		
RSBi (RR/Vt (L)) *	342.1 (236.5–457.9)		Rapid Shallow Breathing
RSBi (RR/mL/kg) *	3.9 (3.2–5.4)		
Relative Expired Abdomen (%)	77 (68–90)		Prevalent diaphragmatic component physiological in supine position
Relative Expired Chest (%)	23 (10–32)		
Chest contribution to Vt (%)		59 CI 95% (45.4–72.6) age 2.3 [28]	
Phase Angle (°)	48.7 (41.6–63.2)	11.8 CI 95% (10.2–13.4) age2.9 [28]	Increased thoraco-abdominal asynchrony

* Vt (mL) was calculated by the SLP software by averaging a pre-imposed standard Vt of 8 mL/patients’ weight in kg entered manually for the number of breaths within the time of recording.

## Data Availability

Anonymised data presented in this study are available on request from the corresponding author.
